# The Histone Chaperones Nap1 and Vps75 Bind Histones H3 and H4 in a Tetrameric Conformation

**DOI:** 10.1016/j.molcel.2011.01.025

**Published:** 2011-02-18

**Authors:** Andrew Bowman, Richard Ward, Nicola Wiechens, Vijender Singh, Hassane El-Mkami, David George Norman, Tom Owen-Hughes

**Affiliations:** 1 Wellcome Trust Centre for Gene Regulation and Expression, College of Life Sciences, University of Dundee, Dundee DD1 5EH, UK; 2 Nucleic Acids Structure Research Group, College of Life Sciences, University of Dundee, Dundee DD1 5EH, UK; 3 School of Physics and Astronomy, University of St. Andrews, St. Andrews FE2 4KM, UK

## Abstract

Histone chaperones physically interact with histones to direct proper assembly and disassembly of nucleosomes regulating diverse nuclear processes such as DNA replication, promoter remodeling, transcription elongation, DNA damage, and histone variant exchange. Currently, the best-characterized chaperone-histone interaction is that between the ubiquitous chaperone Asf1 and a dimer of H3 and H4. Nucleosome assembly proteins (Nap proteins) represent a distinct class of histone chaperone. Using pulsed electron double resonance (PELDOR) measurements and protein crosslinking, we show that two members of this class, Nap1 and Vps75, bind histones in the tetrameric conformation also observed when they are sequestered within the nucleosome. Furthermore, H3 and H4 trapped in their tetrameric state can be used as substrates in nucleosome assembly and chaperone-mediated lysine acetylation. This alternate mode of histone interaction provides a potential means of maintaining the integrity of the histone tetramer during cycles of nucleosome reassembly.

## Graphical abstract

**Graphical Abstract:**

**Figure:**
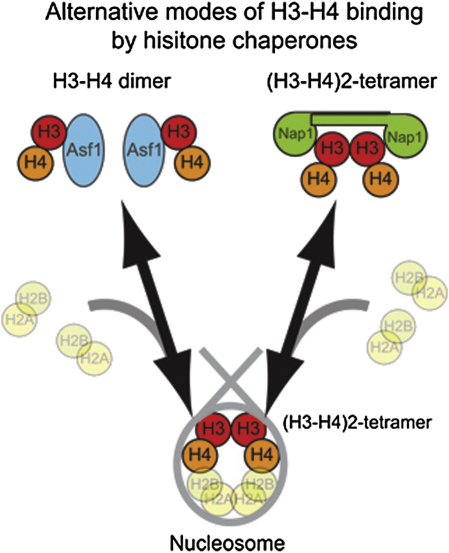

**Highlights:**

► Site-specific crosslinking of H3 shows Nap proteins bind a tetramer of H3 and H4 ► Tetrameric conformation confirmed by EPR measurements and in vivo crosslinking ► Nap1 can deposit a whole (H3-H4)_2_ tetramer onto DNA ► Vps75-Rtt109 preferentially acetylates tetrameric H3 and H4

## Introduction

Access to the genetic information of eukaryotic organisms is regulated in part by the assembly and disassembly of chromatin. This involves interplay between the action of histone chaperones, ATP-dependent remodeling enzymes, and histone-modifying enzymes.

There are at least seven major structural families of histone chaperones related to nucleoplasmin, chromatin assembly factor 1 (CAF-1), antisilencing factor 1 (Asf1), facilitates chromatin transcription (FACT), regulator of Ty1 transposition 106 (Rtt106), chaperone for Htz1/H2A-H2B dimer (Chz1), and nucleosome assembly protein 1 (Nap1) (Das et al., 2010; Hansen et al., 2010). High-resolution crystal structures have been obtained for a number of histone chaperones including Asf1 (Daganzo et al., 2003; Mousson et al., 2005), Vps75 (Berndsen et al., 2008; Park and Luger, 2008; Tang et al., 2008), nucleoplasmin (Dutta et al., 2001), and Nap1 (Park and Luger, 2006b). However, only for Asf1 is there detailed information regarding the nature of the interaction between a chaperone and the histones it binds. In this case, nuclear magnetic resonance has been used to show that the C-terminal helix of H3 interacts with a concave groove on Asf1 (Mousson et al., 2005; reviewed by Henikoff, 2008). Subsequently, Asf1 has been cocrystallized with the histone fold domains of histones H3 and H4 (English et al., 2006; Natsume et al., 2007). This structure indicates that Asf1 makes contacts with both H3 and H4, and, consistent with previous biochemical investigations (English et al., 2005), these histones are present as a dimer rather than tetramer. Furthermore, the interaction of Asf1 with the C terminus of H3 obscures an interaction surface required for formation of a (H3-H4)_2_ tetramer. Further support for the splitting of the tetramer has come from pull-down experiments suggesting that the Asf1-H3-H4 heterotrimer prevails in complex with CAF-1 and HIRA in mammalian cells (Tagami et al., 2004).

Although these observations all support the splitting of histone tetramers after association with histone chaperones, there is historic literature (reviewed in Annunziato, 2005), supported by recent studies (Xu et al., 2010), indicating that the integrity of a substantial proportion of histone tetramers is maintained after DNA replication. These observations are difficult to reconcile with the splitting of the (H3-H4)_2_ tetramer when associated with chaperones, as has been proposed to occur during chromatin assembly and disassembly reactions. One possible means of reconciling the persistence of the parental tetramer is that the mode of interaction between alternate classes of histone chaperone and their cargo differs. Importantly, interactions between Nap1 related chaperones have not been characterized in as much detail as has been possible for Asf1.

*S. cerevisiae* has at least two Nap proteins, Nap1 and Vps75. Crystal structures of these two chaperones show that they exist as tightly bound homodimers adopting the so-called “headphone” fold (Berndsen et al., 2008; Park and Luger, 2006b; Park et al., 2008b; Tang et al., 2008; Tsubota et al., 2007). Nap1 and Vps75 bind all four core histones with nanomolar affinity in vitro (Andrews et al., 2008; Berndsen et al., 2008; Park et al., 2008b; Tsubota et al., 2007). Nap1 has been shown to associate with all four core histones in vivo (Krogan et al., 2006), with Vps75 being found in complex with the acetyl transferase Rtt109 in vivo, where it aids in acetylation of newly synthesized histones (Fillingham et al., 2008; Li et al., 2008; Selth and Svejstrup, 2007; Tsubota et al., 2007), possibly assisting histone deposition during S phase (Kaplan et al., 2008; Li et al., 2008). Nap1 has roles in histone transport into the nucleus (Chang et al., 1997; Mosammaparast et al., 2001) and acts in conjunction with ATP-dependent chromatin remodeling factors in the assembly and disassembly of nucleosomes in vitro (Lorch et al., 2006; Tyler et al., 1999).

Initially, it was reported that a Nap1 dimer binds a single H3-H4 dimer (McBryant et al., 2003; Tóth et al., 2005). These calculations were derived from observing complex formation under native polyacrylamide gel electrophoresis, an approach that is often prone to error, especially with a self-associating system (Park et al., 2008a; Tóth et al., 2005). More recent studies using a fluorescent binding assay to analyze the thermodynamics of histone-Nap1/Vps75 interactions clearly show that two H3-H4 dimers bind a single Nap1/Vps75 dimer (Andrews et al., 2008, 2010; Park et al., 2008b). However, with these approaches, it was not possible to distinguish whether histones H3 and H4 are bound as two dimers or one tetramer. The presence of an exposed beta sheet face within the earmuff domain of Nap proteins has led some to suggest, guided by homology modeling to Asf1, that binding may occur via splitting of the tetramer, one H3-H4 dimer binding per beta sheet face in a similar fashion to Asf1 (Gill et al., 2009).

Here, we apply protein crosslinking and site-directed spin labeling (SDSL) with pulsed electron double resonance (PELDOR) to probe the conformation of H3 and H4 when in complex with Nap1, Vps75, and Asf1. Consistent with previous reports, we find that association with Asf1 causes dissociation of the (H3-H4)_2_ tetramer, resulting in binding of a single H3-H4 dimer. In contrast, histones H3 and H4 bound to Nap1 or Vps75 remain associated as a tetramer, demonstrating that a histone chaperone is capable of binding H3 and H4 in their nucleosomal conformation. Furthermore, we show H3 and H4 remain as a tetramer during deposition onto DNA by Nap1 and that Vps75, but not Asf1, preferentially stimulates Rtt109 directed acetylation of H3 and H4 locked in their tetrameric conformation. The prevalence of a tetramer of H3 and H4 when bound to a histone chaperone potentially provides a molecular explanation as to how the integrity of the histone tetramer is maintained during the course of DNA replication.

## Results

### The Interactions of Asf1 and Nap1 with Histones H3 and H4 Display Contrasting Sensitivity to Ionic Conditions

Within the nucleosome, the predominant mode of histone-DNA interaction is electrostatic: histones present a basic ramp around which the acidic DNA molecule wraps. Outside of the nucleosome, for instance during histone shuttling into the nucleus, the basic surfaces of the histones have to be shielded from spurious interactions with other cell components. Histone chaperones therefore have been defined as proteins that prevent such nonspecific interactions from occurring, promoting proper chromatin assembly (Andrews et al., 2010; Laskey et al., 1978). As a first step toward comparing the mode by which Asf1 and Nap1 interact with H3 and H4, we monitored complex formation during gel-filtration chromatography under different ionic conditions.

**Figure 1:**
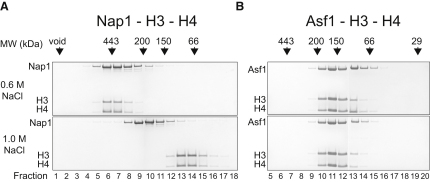
Histone Binding to Nap1, but Not Asf1, Shows Sensitivity to Ionic Strength (A) The elution profiles of Nap1-H3-H4 from gel filtration shown as consecutive fractions separated by SDS-PAGE. Complex formation under 0.6 M NaCl and 1.0 M NaCl was monitored. Fraction numbers are denoted at the bottom of the gel and the elution points of molecular weight markers shown above. (B) The same experiment carried out for Asf1 in complex with H3 and H4.

If Nap1 binds H3 and H4 in a similar fashion to Asf1 (i.e., via the β sheet face within the earmuff domain) we reasoned its interaction should display similar properties. In 0.6 M sodium chloride, both Nap1 and Asf1 elute as higher molecular weight complexes consisting of the chaperone, H3, and H4 (Figures 1A and 1B, top, fractions 6–8 and 10–13, respectively). However, in the high ionic environment of 1 M sodium chloride, H3 and H4 no longer stably associate with Nap1 (Figure 1A, bottom, Nap1 fractions 8–11, H3 and H4 fractions 13–15). In contrast, the complex between Asf1 and histones H3 and H4 remains stable (Figure 1B, bottom). The robustness of the Asf1-histone complex to ionic conditions is likely to result from interactions with the largely hydrophobic dyad interface of H3 (English et al., 2006; Natsume et al., 2007). The sensitivity of the Nap1 histone complex to salt conditions is more reminiscent of the interaction of histones with DNA and suggests a different mode of contact involving ionic interactions. One means by which this could be achieved is if the hydrophobic surfaces of H3 and H4 are sequestered in their tetrameric conformation (observed within the nucleosome), leaving the positively charged DNA binding surfaces available for interactions with Nap1. This led us to hypothesize that the sensitivity of the Nap1-H3-H4 complex to ionic strength, over the Asf1-H3-H4 complex, results from the tetramerization of H3 and H4.

### Targeted Crosslinking of H3 Suggests that Nap1 Binds a Constitutive (H3-H4)_2_ Tetramer

**Figure 2:**
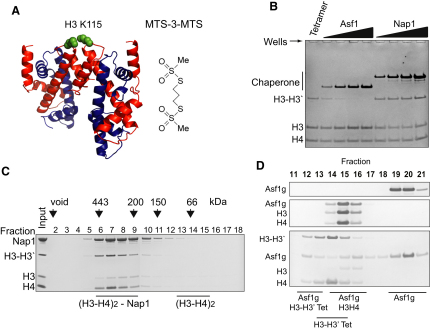
Directed Sulfhydryl-Reactive Crosslinking Suggests that Nap1 Binds H3 and H4 in Their Tetrameric Conformation (A) The structure of the (H3-H4)_2_ tetramer from the nucleosome crystal structure showing the proximity of H3 K115 residues (green spheres) (image created with Pymol, http://www.pymol.org/). Chemical structure of the compound MTS-3-MTS used to crosslink cysteines engineered at position 115 is shown alongside. (B) The extent of crosslinking under increasing concentrations of cysteine free Nap1 or Asf1 shown by separation of the reaction components by SDS-PAGE. Concentration of chaperone from left to right was 0.25, 0.5, 1.0, and 2.0 μM monomer. (C) Gel-filtration analysis of the H3 K115C crosslinked tetramer in the presence of Nap1 showing that they remain stably associated under the conditions used. (D) Globular Asf1 was mixed with noncrosslinked (middle) and crosslinked (bottom) tetramer and the complexes separated by gel-filtration chromatography. The majority of Asf1 does not form a complex with crosslinked tetramers. See also Figures S1 and S2.

To test the hypothesis that Nap1 can bind H3 and H4 in their tetrameric conformation, we developed a cysteine crosslinking approach to report on the tetramerization of H3 and H4. Using the 1.9 Å nucleosome structure as a guide (Richmond and Davey, 2003), we scouted for residues on H3 that are within a few Angstroms of each other yet not involved in the H3-H3′ interaction. We identified H3 K115 as a potential crosslinking site and engineered a cysteine at this location (Figure 2A). The Cβ atoms of this residue are within 9 Å of each other; therefore, formation of a tetramer should result in crosslinking at this position in the presence of a homobifunctional thiol-reactive crosslinker. This crosslinked, H3-H3′ species can then be easily resolved from noncrosslinked H3 by SDS-PAGE. A methanethiosulfonate crosslinker with a 3 carbon spacer (termed MTS-3-MTS, Figure 2A) was found to be most efficient (data not shown). With a 1:1 ratio of tetramer to MTS-3-MTS, crosslinking occurred rapidly and was all but complete by 1 min, resulting in 56% of total H3 crosslinked at 1 μM complex (Figures S1A and S1B available online). Importantly, the gel-filtration profile of crosslinked tetramer was unchanged indicating that H3 crosslinks occurred specifically within the tetramer rather than between different H3-H4 tetramers (Figure S1C).

Using this crosslinking approach, we probed the conformation of H3 and H4 when bound to Asf1 and Nap1. Asf1 has been shown to disrupt the histone tetramer by binding to the C terminus of H3, obscuring the H3-H3′ interface (English et al., 2005, 2006; Natsume et al., 2007). Therefore, one would expect that Asf1 would inhibit crosslinking in our assay. Indeed, this is exactly what we see (Figure 2B). Titrating cysteine free Asf1 against the (H3-H4)_2_ tetramer prior to crosslinking, we see a gradual disappearance of the H3-H3′ crosslinked species and an increase in noncrosslinked H3. In contrast to this, when a cysteine free form of Nap1 (Andrews et al., 2008) is titrated into the reaction prior to crosslinking the H3-H3′ crosslinked species persists (Figure 2B), suggesting that the H3-H3′ interface is retained when bound to these chaperones. The final titration point for Nap1 was subject to gel-filtration chromatography (Figure 2C) showing that crosslinked H3 remains stably associated with Nap1. Furthermore, precrosslinked tetramers formed complexes with Nap1 that were indistinguishable from those formed in the absence of crosslinking (compare Figure S2 and Figure 1A). This indicates that crosslinking does not markedly affect the Nap1-H3-H4 interaction.

In contrast to Nap1, the ability of Asf1 to bind histones H3 and H4 was impaired if they were first crosslinked (Figure 2D). As Asf1, Asf1-H3-H4, and the (H3-H4)_2_ tetramer all elute in similar fractions after gel-filtration chromatography, a globular form of Asf1 (Asf1g) (English et al., 2005) comprising the histone binding domain was used to aid in the separation of complexes as it elutes in later fractions (Figure 2D, fractions 19–21). In the absence of crosslinker, Asf1g migrates with the histones in a heterotrimeric complex (Figure 2D, fractions 14–16). In contrast to this, in the presence of crosslinked tetramer Asf1g's ability to bind histones is severely impaired. The majority of Asf1g elutes as a monomer (Figure 2D, fractions 19–21) with a small amount eluting with residual noncrosslinked tetramer (fractions 14–16) and a smaller fraction eluting with crosslinked tetramer (fractions 12 and 13). This is consistent with the crosslink acting to stabilize H3 and H4 in a nucleosomal conformation disfavored during Asf1 binding. However, it is important to note that this stabilization is not necessarily absolute. Some reorganization of tetramer structure may still be possible, despite the presence of the crosslink. This could account for the residual binding of Asf1 to crosslinked H3. Overall, these observations demonstrate that our crosslinking approach is both an excellent reporter on tetramer structure and an effective method of stabilizing the (H3-H4)_2_ tetramer.

### Long-Range Distance Measurements between Histones Indicate that H3 and H4 Exist as a Tetramer When Bound to Nap1

**Figure 3:**
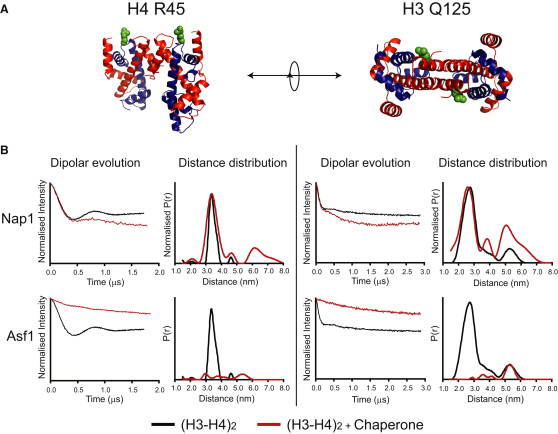
Extraction of Long-Range Distances between Spin-Labeled H3-H4 Dimers Agrees with a Tetrameric Conformation When Bound to Nap1 (A) Positions of spin labels on H4 R45 and H3 Q125 (green spheres) are shown on the structure of the tetramer taken from the nucleosome (PDB code 1KX5, images created with Pymol, http://www.pymol.org/). (B) The background corrected dipolar evolution and the distance distribution for each labeling site in complex with Nap1 and Asf1 is shown (red trace). For comparison the tetramer value extracted from the tetramer alone is shown alongside (black trace). See also Figure S3.

Our cysteine-reactive crosslinking approach demonstrated that position K115C on H3 and H3′ are in close proximity when bound to Nap1. Although this strongly suggests that this is the result of (H3-H4)_2_ tetramer formation, the possibility still exists that H3 and H4 may be bound as two separate dimers in an orientation where K115C of H3 and H3′ are within crosslinking distance. Thus, to further test our hypothesis that H3 and H4 are in their tetrameric conformation, we used SDSL and PELDOR to extract distance measurements from H3 and H4 when in complex with the chaperones Nap1 and Asf1. This involves attaching nitroxide reporters via engineered cysteine residues and extracting distance information from spin-spin dipole interactions observed after pulsed microwave radiation. This approach was previously used to characterize the structure of the (H3-H4)_2_ tetramer and identified labeling sites on both H3 and H4 that give discrete distance distributions in close agreement with distances calculated from the nucleosome crystal structure (Bowman et al., 2010). These sites, H4 R45R1 and H3 Q125R1 (Figure 3A), were used to probe the structure of the (H3-H4)_2_ tetramer under conditions established to result in stable complexes with Nap1 and Asf1.

In these experiments three possible outcomes were anticipated. First, if a (H3-H4)_2_ tetramer is bound then the distance distributions from a complex of proteins should be comparable to that of free tetramer. Second, if two H3-H4 dimers are bound in a nontetrameric conformation, an alternative distance distribution should be observed. Third, if the tetramer is split with just a single H3-H4 dimer being bound, as has been suggested (McBryant et al., 2003; Tóth et al., 2005), there would be no spin-spin dipolar coupling, and thus no distance.

It can be seen for both labeling sites, H4 R45R1 and H3 Q125R1, that the distance distribution corresponding to a tetramer of H3 and H4 persists when the histones are in complex with Nap1 (Figure 3B). It is also notable that minor distance distributions increase in the presence of histone chaperones. The conversion of PELDOR data to distance distributions by Tikhonov regularization is prone to background derived errors. This is often observed as minor apparent distance distributions at longer distances (Bowman et al., 2010). Adjustment of background correction parameters results in apparent movement of artifactual distance distributions but does not have a large effect on real distance distributions. This is illustrated in Figure S3. For this reason, we believe that the major distance distributions are significant whereas the minor ones are not. In contrast, formation of Asf1-H3-H4 complexes completely ablates the dipolar coupling, and this is observed in the time trace as an unmodulated exponential decay (Figure 3B). Thus, in addition to the short-range interaction between H3 K115C identified by crosslinking, longer-range EPR measurements indicate that a tetrameric configuration predominates in Nap1-H3-H4 complexes. We conclude from this that H3 and H4 are bound to Nap1 in a tetrameric conformation very similar to that observed within nucleosomes (Bowman et al., 2010) (Figure S3).

### The Nap1-Related Chaperone Vps75 Also Binds H3 and H4 as a Tetramer

**Figure 4:**
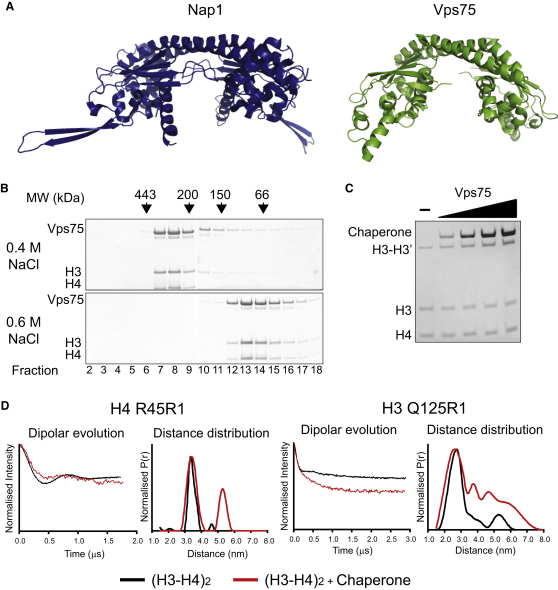
Vps75 Binds H3 and H4 in Their Tetrameric Conformation (A) Nap1 (PDB code: 2ZR2) and Vps75 (PDB code: 3C9D) both adopt the headphone fold. Structures shown for comparison (images created with Pymol, http://www.pymol.org/). (B) H3 and H4 binding by Vps75 displays sensitivity to ionic strength. Fractions from gel-filtration chromatography are shown under 0.4 M and 0.6 M sodium chloride. Vps75-H3-H4 form a complex at 0.4 M sodium chloride (fractions 7–9, top panel), but elute in their separate fractions at 0.6 M (note that both free Vps75 dimer and H3-H4 tetramer elute in fractions 13–15). (C) Histone tetramer-specific crosslinking of H3 K115C by MTS-3-MTS persists in the presence of cysteine free Vps75. Concentration of Vps75 from left to right was 0.25, 0.5, 1.0, and 2.0 μM monomer. (D) Long-range distance from histone tetramer spin-labeled at position H4 R45R1 and H3 Q125R1 complexed with Vps75 were extracted with the PELDOR experiment. Background corrected dipolar evolutions and distance distributions for each are shown (red trace). For comparison, distances measured for the tetramer alone are shown alongside (black trace).

Differences in the structures of Nap1 and Asf1 are likely to confer the different modes of histone interaction we have detected for these two proteins. Sequence alignments indicate that Nap1-related proteins are broadly conserved through evolution and that many eukaryotes encode multiple Nap1-related proteins (Park and Luger, 2006a). For example, budding yeast encode the protein Vps75, whose structure is closely related to that of Nap1 (Figure 4A). This raises the possibility that the interaction with tetrameric H3-H4 may be a general feature of Nap1-related histone chaperones. To investigate this further, we set out to characterize the interaction of histones H3 and H4 with Vps75.

Just as we had previously observed for Nap1, Vps75 formed complexes with histones H3 and H4 that were sensitive to ionic conditions, though in this case 0.6 M sodium chloride was sufficient to cause dissociation (Figure 4B) with a stable association occurring at 0.4 M sodium chloride. Crosslinking at H3 K115C was not reduced when Vps75 was bound to tetramers (Figure 4C) and crosslinking was retained in the complexes when assessed by gel filtration (data not shown). Finally, PELDOR measurements between H4 R45R1 and H3 Q125R1 indicate that a tetrameric configuration is retained within these complexes (Figure 4D). From these observations, we conclude that Vps75, like Nap1, binds to histones H3 and H4 in a tetrameric configuration, suggesting that tetramer binding may be a general property of Nap proteins.

### In Vivo Evidence for the Association of Histone Chaperones with Histones in a Tetrameric Conformation

Asf1 and Vps75 are known chaperones for H3 and H4, whereas Nap1 is typically regarded as an H2A-H2B chaperone, despite its high affinity for H3 and H4 in vitro (Andrews et al., 2008). In contrast with this view, a high-throughput coimmunoprecipitation study has revealed a high-scoring physical interaction with all four core histones (Krogan et al., 2006). To further validate the Nap1-H3-H4 interaction in vivo, we isolated Nap1 by tandem affinity purification (TAP) (Puig et al., 2001) and then immunoblotted for histones. H2A and H2B are the major histones associating with Nap1 (Figure S4A), an observation that may in part relate to the function of Nap1 as a nuclear import factor for H2A and H2B but not H3 or H4 (Chang et al., 1997; Mosammaparast et al., 2001). However, in addition, we could detect H4-associated with Nap1 (Figure S4A), suggesting that Nap1 is likely to form complexes with all histones in vivo. We next generated a strain in which the sole source of H3 was myc tagged and included the H3 K115C mutation we had used in vitro. This strain was viable with no growth defects detected (data not shown). TAP tagging the chaperones Asf1, Nap1, and Vps75 in this background provided a means of recovering the H3 associated with each chaperone. We next sought to monitor the conformation of histones associating with these different chaperones by crosslinking.

Initial experiments using chemical crosslinkers such as MTS-3-MTS and bis-maleimidoethane (BMOE) resulted in low levels of H3-H3′ crosslinking and the appearance of additional high-molecular-weight crosslinked species. When using these compounds with recombinant proteins, we had observed that the stoichiometry of crosslinker to cysteines was critical and likely to be more difficult to control using native proteins. Instead, we pursued an alternative crosslinking approach whereby disulphide bond formation between juxtaposed cysteine residues is catalyzed by copper(II) chelated with 1,10-phenanthroline (CuP) (Kobashi, 1968). Crosslinking with CuP gave similar results to chemical crosslinkers in vitro (Figure S4C) but did not display the same sensitivity to stoichiometry, with crosslinking efficiency increasing with CuP concentration, as previously reported (Kobashi, 1968).

**Figure 5:**
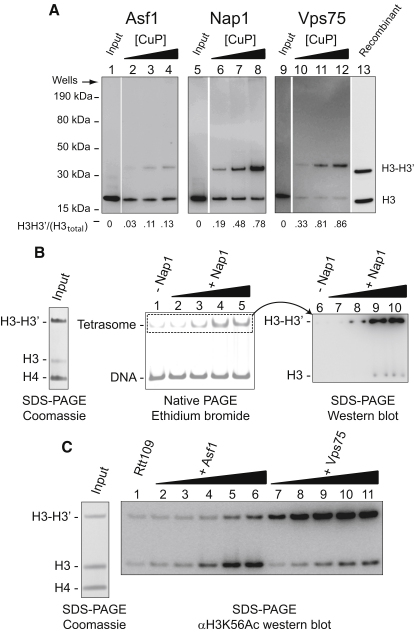
Nap1 and Vps75 Copurify with Tetrameric H3-H4 and Can Utilize H3 and H4 Trapped in Their Tetrameric Conformation for Their Biological Functions In Vitro (A) TAP-tagged Asf1, Nap1, and Vps75 were purified from strains expressing H3K115C-myc as the sole source of histone H3. Complexes were subject to crosslinking in the presence of 0.064, 0.25, or 1.0 mM CuP (Asf1, lanes 2–4; Nap1, lanes 6–8; Vps75, lanes 10–12), separated by SDS-PAGE, and immunoblotted within an anti-myc antibody. The intensity of the anti-myc signal was quantified, and the proportion of crosslinked H3 of the total is displayed at the bottom of each lane. Lane 13 contains recombinant partially crosslinked (nontagged) H3 developed with a H3 antibody as a size standard. (B) (H3-H4)_2_ tetramer deposition by Nap1. Left: Crosslinked tetramer input stained with Coomassie. This indicates that the majority of histone H3 present is crosslinked. Center: Monotetrasomes were separated from free DNA by native gel electrophoresis. Lane 1, without Nap1; lanes 2, 3, 4, and 5, 0.2, 0.5, 1.0, and 2.0 μM Nap1, respectively. Right: The monotetrasomal bands were excised from the native gel, subjected to SDS-PAGE, and probed by western blotting for H3. Lanes 6–10 correspond to lanes 1–5 from the center panel. This shows that crosslinked tetramers are present in tetrasomes assembled by Nap1. (C) Vps75 preferentially promotes Rtt109 acetylation of crosslinked tetramer, whereas Asf1 preferentially directs Rtt109 acetylation in favor of noncrosslinked tetramer. Left: Equimolar amounts of crosslinked to noncrosslinked H3 K115C was used as an input. Right: Lane 1, Rtt109 alone (140 nM); lanes 2–6, 60, 80, 100, 120, and 140 nM each of Asf1 and Rtt109, respectively; lanes 7–11, 10, 15, 20, 25, and 30 nM of (Vps75)_2_-Rtt109 complex, respectively. See also Figure S4.

Using this approach, we found crosslinking of H3 K115C-myc associated with Nap1 and Vps75 to be considerably more efficient than H3 bound by Asf1 (Figure 5A). Indeed, 78% of Nap1 and 86% of Vps75-associated H3K115C-myc was crosslinked at 1 mM CuP, whereas only 13% of Asf1-associated H3K115C-myc was crosslinked at the same CuP concentration. The minor crosslinked fraction in the Asf1 pull-down may result from an equilibrium between (H3-H4)_2_ and Asf1-H3-H4 at the low concentrations used in the crosslinking experiment or the ability of some Asf1 to associate with H3 despite the presence of the crosslink as observed in vitro (Figure 2D). For verification of the size of the crosslinked H3, recombinant H3 was run alongside (Figure 5A). The lack of the myc tag means that the recombinant H3 and crosslinked H3 migrate slightly faster than their tagged counterparts (Figure 5A).

### Nap1 Facilitates the Loading of Intact H3-H4 Tetramers onto DNA

Having established that Nap1 and Vps75 can bind a tetramer of H3-H4, we wanted to see whether these chaperones could utilize H3-H4 trapped in their tetrameric conformation using functional assays. The observation that Asf1 causes a structural rearrangement within H3 and H4 has been used to propose a model in which tetramer splitting is an obligate step in the action of Asf1 in the assembly and disassembly of nucleosomes (English et al., 2006; Natsume et al., 2007). To investigate whether Nap1 is able to deposit tetrameric H3-H4 complexes onto DNA we prepared tetramers that were crosslinked at H3 K115C to an efficiency of 80% (Figure 5B). This improved crosslinking efficiency was achieved by crosslinking at 10 μm rather than 1 μm. Deposition of H3 and H4 onto DNA to form a tetrasome is the first step in nucleosome assembly. To isolate this first stage of nucleosome assembly, we used a 91 bp DNA fragment (termed −28W−28) derived from the well-defined 601 positioning sequence (Thåström et al., 1999). This size fragment allows only a single tetramer to be assembled onto it, migrating as a single super-shifted band upon native gel electrophoresis, and thus allowing for unambiguous assignment. Assembly of crosslinked tetramers onto the 91 bp DNA fragment was inefficient in the absence of Nap1 (Figure 5B, lane 1), but enhanced in the presence of Nap1 (Figure 5B, lanes 2–5). In order to investigate whether the tetramers transferred onto DNA by Nap1 retained a tetrameric conformation, and were not derived from the uncrosslinked population, we excised the tetrasomal band, resolved the histones by SDS-PAGE, and assessed the crosslinking status of H3 after western blotting. Figure 5B, lanes 6–10, confirms that the majority of the H3 assembled onto DNA was crosslinked, suggesting that dissociation of the histone tetramer is not an obligate step in nucleosome assembly directed by Nap1.

### Vps75 Presents a Tetramer of H3-H4 to Rtt109 for Acetylation

The majority of Vps75 in vivo is found in complex with the acetyl transferase Rtt109, where it is proposed to enhance its acetyl transferase activity (Fillingham et al., 2008; Selth and Svejstrup, 2007; Tsubota et al., 2007). Asf1 also regulates the activity of Rtt109 (Chen et al., 2008; Fillingham et al., 2008; Li et al., 2008; Tsubota et al., 2007), and both enzymes have been shown to stimulate Rtt109's activity in vitro (Berndsen et al., 2008; Tsubota et al., 2007). Thus, we reasoned that if Vps75 binds a tetramer of H3 and H4, the Vps75-Rtt109 complex should acetylate crosslinked tetramer as efficiently as noncrosslinked tetramer. On the contrary, one would expect acetylation of H3 by an Asf1-Rtt109 complex to be diminished when using crosslinked tetramer as a substrate. To test this, we used a competition assay in which half of the H3 was in a crosslinked tetramer and half was in a noncrosslinked tetramer. Rtt109 on its own was very poor at acetylating H3 (probed by an antibody to acetylated H3 K56) (Figure 5C, lane 1). Addition of Asf1 increased the efficiency of acetylation by Rtt109 largely on noncrosslinked H3, as expected (Figure 5C, lanes 2–6). In contrast to this, acetylation of H3 by Vps75-Rtt109 was directed predominantly toward crosslinked (H3-H4)_2_ tetramer (Figure 5C, lanes 7–11). This suggests that Vps75 not only tolerates a tetramer of H3 and H4, but its chaperoning activity is actually enhanced by the stabilization of the tetramer. Indeed, at physiological ionic strength, in which the acetylation reactions were carried out, the interaction between H3-H3′ is weakened, resulting in an equilibrium between H3-H4 dimers and (H3-H4)_2_ tetramers (Baxevanis et al., 1991). Therefore, the preference we see toward acetylation of H3 crosslinked at K115C may be due to the stabilizing effect that this crosslink has on the tetramer at these salt concentrations.

### Nap1 Can Restore the Tetrameric Conformation of a Nontetramerizing Mutant

**Figure 6:**
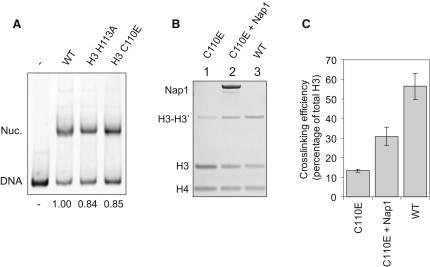
Nap1 Stabilizes the (H3-H4)_2_ Tetramer (A) Histones carrying nontetramerizing mutations can form bona fide nucleosomes. The positions of DNA and nucleosomes (Nuc.) are indicated. The efficiency of nucleosome assembly, quantified by the proportion of DNA incorporated over total DNA present, is displayed at the bottom of the gel as a fraction of the wild-type. (B) Nap1 can partially restore tetramerization of H3 C110E-containing dimers. H3 K115C crosslinking mutation was introduced into the C110E construct and crosslinked alone and in the presence of Nap1. Crosslinking efficiency of the wild-type (containing H3 K115C) is shown for comparison. (C) Quantification of crosslinking efficiency shown in (B). The average and standard deviation of three independent experiments are shown. See also Figure S5.

The ability of Nap1 and Vps75 to utilize crosslinked H3 suggests that they may act to stabilize the tetrameric conformation of H3 and H4. To investigate this, we made use of previously identified mutations in the tetramerization interface that shift the tetramer-dimer equilibrium to favor the dimeric species. A H3 H113A mutation removes a key interaction with H3′ D123A (Andrews et al., 2008), whereas a H3 C110E mutation introduces a charge repulsion (Banks and Gloss, 2004). Consistent with these reports, we find the elution volumes of H3-H4 carrying mutations H3 H113A or H3 C110E are increased when compared to the wild-type at 0.2 M sodium chloride (Figure S5). Although these mutations shift the equilibrium of free H3 and H4 in favor of the dimeric form, these mutants were still capable of forming particles with an electrophoretic mobility identical to that of wild-type nucleosomes upon salt deposition (Figure 6A). This suggests that in the presence of binding partners the nontetramerizing mutations H3 H113A and H3 C110E are subdued. This led us to investigate whether the interaction with histone chaperones would also stabilize the tetramer. To test this, we introduced the H3 C110E nontetramerizing mutation into the H3 K115C crosslinking construct. As expected, the nontetramerizing mutation resulted in a significant decrease in crosslinking efficiency compared to wild-type H3 (both containing K115C mutations) (Figure 6B, compare lanes 1 and 3). Upon addition of Nap1 to the reaction, crosslinking efficiency was improved markedly (Figure 6B, compare lanes 1 and 2), resulting in a partial yet significant rescue of the tetrameric species (Figures 6B and 6C). These data suggest that Nap1 can interact with mutant histones with a weakened tetramerization interface (Andrews et al., 2008) and in doing so can restore their tetrameric conformation.

## Discussion

Histone chaperones have been defined on the basis of their ability to bind histones and affect chromatin metabolism without the requirement for ATP hydrolysis. Although they all share this function, crystallographic analysis shows that histone chaperones adopt diverse tertiary structures, suggesting they may harbor alternate modes of histone binding. Understanding these interactions at a structural and biochemical level is likely to give key insights into their biological function.

Both site-directed crosslinking and PELDOR measurements indicate that histones H3 and H4 adopt a tetrasomal configuration related to that observed in the nucleosome when associated with the histone chaperones Nap1 and Vps75. However, we cannot rule out the possibility that under some circumstances, for example in the presence of limiting histones, that dimeric H3-H4 could be bound. Nonetheless, we have found that these Nap proteins retain H3-H4 in their tetrameric conformation when purified from yeast and can utilize H3 and H4 locked in their tetrameric conformation as substrates in assays related to their biological functions: Nap1 is able to deliver a crosslinked histone tetramer to DNA, and Vps75 preferentially enhances the activity of Rtt109 for tetrameric H3-H4.

A recent report demonstrated that histone acetylation directed by Rtt109 in the presence of Vps75 was only slightly less efficient when mutant histones that display reduced tetramerization were used (Kolonko et al., 2010). This was interpreted as evidence that the Vps75-Rtt109 complex acetylates dimeric histones H3 and H4. However, our observations suggest that H3 C110E can adopt a tetrameric conformation either after assembly into nucleosomes or incubation with Nap1. It appears to be possible that Vps75 may also act to restore these mutant histones to a tetrameric conformation. This illustrates a limitation in the use of histone mutants to report on the dimer tetramer interface, which may be more generally applicable. Although these mutations favor dissociation of (H3-H4)_2_ tetramers in solution, this is not necessarily the case in larger complexes that may provide a scaffold capable of restoring tetramerization.

As well as being able to bind H3 and H4 in their tetrameric conformation, both Nap proteins analyzed in this study show a strong dependency on the ionic environment for their interaction, which has also been observed for Nap1's interaction with H2A-H2B dimers (Park and Luger, 2006b). Thus, in some respects, Nap1 and Vps75 seem to act as a functional DNA mimic. All Nap proteins crystallized to date have shown a distinct charge distribution with the cleft formed between the two earmuff domains being highly acidic. This could be a possible interaction site for the basic ramp of the histone tetramer, and indeed, after removing the H3 αN helix, which is likely to be flexible in solution (Bowman et al., 2010), the basic geometry would allow binding in this manner. In this way, Nap1 may function to drive nucleosome assembly by presenting a tetramer of H3 and H4 to DNA in a manner that is conducive to nucleosome formation.

Our observations indicate that Nap1-related histone chaperones have the properties required to shuttle histone tetramers off and on DNA without a requirement for separating H3-H4 dimers. This is consistent with both historical and recent observations indicating that after DNA replication, the majority of maternal H3 and H4 dimers do not mix with nascent histones (Jackson, 1990; Prior et al., 1980; Xu et al., 2010). In contrast, it has previously been shown that Asf1 interacts with histones H3 and H4 in a dimeric conformation. This may represent a normal step in the delivery of newly synthesized histones into chromatin assembly pathways and also contribute to replication independent chromatin assembly where tetramer splitting has been observed to be more frequent (Xu et al., 2010). The existence of distinct pathways by which different classes of histone chaperone act during chromatin assembly is anticipated to influence the way in which histone modifications and variants are transmitted (Campos et al., 2010). Investigation of the nature of the interaction between histone chaperones and their cargos is likely to provide further insight into this process.

## Experimental Procedures

### Protein Expression and Purification

A detailed description of protein expression and purification procedures can be found in the Supplemental Experimental Procedures. Histones were essentially expressed as described previously (Luger et al., 1997). Cysteine-free versions of Asf1, Nap1, and Vps75 were generated by site-directed mutagenesis, expressed from a pET15b vector (Novagen) in *E. coli*, and purified by cobalt affinity, anion exchange, and gel-filtration chromatography. Rtt109 was expressed from a pET28a vector and purified by cobalt affinity, cation-exchange chromatography, and gel-filtration chromatography. Cysteine-to-alanine mutations were found not to affect interactions with histones. 6His-tagged Vps75 (pET15b vector) and GST-tagged Rtt109 (pGEX 6P1 vector, GE Healthcare) were coexpressed in *E. coli* and purified by sequential cobalt/glutathione affinity and cation-exchange chromatography. Asf1g, consisting of residues 1–164, was cloned by PCR into pET15b and expressed and purified the same as the full length.

### Analytical Gel Filtration

Complexes were analyzed by microgel-filtration chromatography with a Superdex S200 PC 3.2/30 column (GE Healthcare) under the indicated sodium chloride concentrations in 20 mM HEPES-KOH (pH 7.5), 1 mM EDTA, 30% glycerol. Twenty microliters 25 μM complex was loaded on to the column and with spanning the void to bed volume analyzed by SDS-PAGE.

### PELDOR Experiments

Spin labeled histone-chaperone complexes were prepared as described in the Supplemental Experimental Procedures. PELDOR experiments were executed with a Bruker ELEXSYS E580 spectrometer operating at X band with a dielectric ring resonator and a Bruker 400U second microwave source unit. All measurements were made at 50 K with an overcoupled resonator giving a Q factor of approximately 100. The video bandwidth was set to 20 MHz. The four-pulse, dead time-free, PELDOR sequence was used, with the pump pulse frequency positioned at the center of the nitroxide spectrum; the frequency of the observer pulses was increased by 80 MHz. The observer sequence used a 32 ns π pulse; the pump π pulse was in a range of 12–18 ns. The experiment repetition time was 4.08 ms, and the number of scans was sufficient to obtain a suitable signal to noise with 50 shots at each time point, e.g., sample Vps75(C→A)-H4R45R1 was run for 904 scans with 159 time points. PELDOR data analysis was carried out as described in the Supplemental Experimental Procedures.

### Sulfhydryl-Reactive Crosslinking

Crosslinking of histones containing the H3 K115C mutation was carried out at 1 μM (tetramer) in 20 μl reactions with histone chaperones at the stated concentrations in 20 mM HEPES-KOH (pH 7.5) and 0.2 M sodium chloride. Details of crosslinking with methanethiosulfonate, maleimide and Cu(II):1,10-phenanthroline_3_ chemistries, in addition to crosslinking methods related to tetrasome assembly and acetyltransferase assays, are included in the Supplemental Experimental Procedures.

### Strain Construction and TAP-Tag Purifications

Detailed methods describing strain construction can be found in the Supplemental Experimental Procedures. For TAP-tag purifications from strains containing myc-tagged H3 K115C, cells were grown at 30°C in 3xYPAD media until mid-log phase was reached. Cells were resuspended in E buffer (20 mM HEPES-KOH [pH 7.5], 150 mM NaCl, 10 % glycerol, 0.05 % Tween-20), frozen in liquid nitrogen, and lysed in a planetary grinder. TAP-tagged complexes were isolated on IgG Sepharose beads (GE Healthcare), washed extensively in E buffer, and released by cleavage of the protein A tag. TAP-tag purifications from strains carrying endogenous histones were essentially the same, except a second purification step with calmodulin affinity resin (Stratagene) was performed. Histone H2A and H4 were probed with antibodies ab13923 and ab10158 (Abcam), respectively.

### Tetrasome Assembly Assay

A DNA fragment derived from the 601 sequence (Thåström et al., 1999) comprising 45 bp each side of the dyad nucleotide was generated by PCR amplification resulting in a 91 bp fragment termed −28W−28. Twenty microliter reactions containing 1 μM crosslinked tetramer, 1 μM −28W−28, 10 mM dithiothreitol, 0.2 M NaCl, 20 mM HEPES-KOH (pH 7.5), and 1 mM EDTA were made. To these reactions, Nap1 was added to a final concentration of 0, 0.1, 0.25, 0.5, and 1 μM dimer. Assembly was allowed to proceed for 2 hr at 25°C, at which point tetrasomes were resolved from free DNA by native PAGE. After staining with ethidium bromide, the tetrasome bands were excised, soaked in 50 mM β-mercaptoethanol, 0.1% sodium dodecyl sulfate for 10 min, and cast within an SDS-PAGE gel. This step ensured that the crosslinked histone tetramer was derived from only the BMOE crosslinked fraction, and not from H3 K115C, which may have disulphide bonded during native gel electrophoresis. Crosslinked tetramer was separated from noncrosslinked tetramer and probed by western blotting with an anti-H3 antibody (ab1791, Abcam).

### Chaperone-Mediated Acetyltransferase Activity Assay

BMOE-crosslinked tetramer on H3 K115C was analyzed by SDS-PAGE and Coomassie staining. Acetyltransferase reactions were performed in 50 mM Tris HCl (pH 7.5) and 100 mM NaCl with 1 mM acetyl CoA. The 10 μl reactions contained 0.5 μM BMOE-treated tetramer and enzymes as stated and were incubated for 2 hr at 37°C. The histone acetyltransferase activity of Rtt109 was analyzed by immunoblotting using an anti-acetyl-Histone H3 (Lys56) antibody (Upstate).

## Note Added in Proof

While this manuscript was being prepared for publication, Tang et al. reported a structure for a Vps75-Rtt109 complex that differs from that of Kolonko et al. (2010) and may be compatible with the binding of a histone tetramer: Tang, Y., Holbert, M.A., Delgoshaie, N., Wurtele, H., Guillemette, B., Meeth, K., Yuan, H., Drogaris, P., Lee, E.H., Durette, C., et al. (2011). Structure of the Rtt109-AcCoA/Vps75 complex and implications for chaperone-mediated histone acetylation. Structure 19, 221–231. Published online January 19, 2011.

## Supplemental Information

Document S1. Supplemental Experimental Procedures and Five Figures
