# Supplementary material for: The Histone Chaperones Nap1 and Vps75 Bind Histones H3 and H4 in a Tetrameric Conformation
Source: Mol Cell. 2011 Feb 18;41(4):398–408. doi: 10.1016/j.molcel.2011.01.025 (PMC3093613; doi:10.1016/j.molcel.2011.01.025)
Supplement: Document S1. Supplemental Experimental Procedures and Five Figures [file mmc1.pdf]

Molecular Cell, *Volume 41*

Supplemental Information

The Histone Chaperones Nap1 and Vps75 Bind Histones H3 and H4 in a Tetrameric Conformation

Andrew Bowman, Richard Ward, Nicola Wiechens, Vijender Singh, Hassane El-Mkami, David George Norman, and Tom Owen-Hughes

Supplemental Experimental Procedures

Protein expression and purification

Xenopus histones were essentially expressed, purified and refolded as described previously (Andrews et al., 2008; Luger et al., 1997). Point mutations for spin labeling and crosslinking sites were introduced using quick-change PCR (Stratagene). Spin labeling of histones was carried out as in (Bowman et al., 2010). Briefly, refolded tetramers containing H3 Q125C and H4 R45C mutations were reduced with 10 mM dithiotheitol (DTT) in refolding buffer (2 M NaCl, 20 mM HEPES-KOH pH7.5, 1 mM EDTA) before being isolated from misfolded aggregates on a 10/300 Superdex S200 gel filtration column (GE Healthcare), which additionally served to remove reducing agent before labeling with a 10 fold molar excess of S-(2,2,5,5-tetramethyl-2,5-dihydro-1H-pyrrol-3-yl)methyl methanesulfonothioate (MTSL). Labelling was carried out for 1 hour at 25 °C. Excess MTSL was removed by dialysis and aliquots were stored at -80 °C. Histone tetramers containing the H3 K115C mutation were purified in the same way except that after gel filtration they were quickly concentrated (Amicon Ultra, Millipore), flash frozen in liquid nitrogen and stored at -80 °C. Aliquots would stay in their reduced state for at least 4 months without showing a decrease in crosslinking efficiency.

S. cerevisiae Vps75 and Asf1 open reading frames were amplified from yeast genomic DNA by PCR and ligated into a modified pET15b vector (Novagen) containing a 6 histidine N-terminal tag with a precision protease cleavage site. The intron in Vps75 was removed by quick-change PCR (Stratagene). Cloned *S. cerevisiae* Nap1 (a kind gift from Karolin Luger) was amplified and ligated into pET15b. Cysteine to alanine mutants of Nap1, Vps75 and Asf1 were generated by quick-change PCR mutagenesis (Stratagene). Cysteine null mutants were found to behave identically to wild type protein, consistent with previous reports (Andrews et al., 2010; Andrews et al., 2008; Park et al., 2008).

Nap1, Vps75 and Asf1 were expressed and purified by the same method. Expression was in BL21 (DE3) pLysS cells (Stratagene) grown in 2YT media until an OD600nm of 0.8 was reached, at which point 0.5 mM isopropyl β-D-1-thiogalactopyranoside was added. Expression was allowed to proceed for 16 hours at 30 °C. Cells were resuspended in Buffer A (20 mM HEPES-KOH pH7.5, 0.1 M NaCl) with the addition of protease inhibitors E64, pepstatin, ABSF and aprotinin, freeze-thawed and sonicated to lyse. Lysates were centrifuged at 40,000 g to remove cell debris and the soluble fraction passed over HisPur cobalt resin (Thermo Scientific). The resin was washed extensively in Buffer A supplemented with 10 mM imidazole and the protein eluted with Buffer A supplemented

with 200 mM imidazole. Eluted protein was loaded onto a 5 ml HiTrap Q anion exchange column (GE Healthcare) and eluted with a linear gradient of 0.1 M to 1.0 M NaCl in Buffer A + 2 mM DTT. Fractions containing Nap1/Vps75/Asf1 were pooled, concentrated and subjected to gel filtration chromatography on a 16/60 S200 column (GE Healthcare) in Buffer B (20 mM HEPES-KOH pH 7.5, 0.5 M NaCl, 1 mM EDTA). Fractions containing Nap1/Vps75/Asf1 were pooled, concentrated on an Amicon Ultra centrifugal concentrator (Millipore) and aliquots stored at -80 °C.

Rtt109 cloned into a pGEX 6P vector (GE Healthcare) was a kind gift from John Rouse. Vps75 and Rtt109 were co-expressed as in BL21 (DE3) cells (Stratagene) after induction at OD600nm of 0.8 with 0.5 mM isopropyl β-D-1-thiogalactopyranoside. After 12 hours expression at 30 °C cells were resuspended in Buffer A supplemented with the aforementioned protease inhibitors, lysed by sonication and sequentially purified on HisPur cobalt resin (Thermo Fisher) and SuperGlu glutathione resin (Generon) to isolate the Vps75-Rtt109 complex. The eluate from the glutathione elution was cleaved with precision protease at 4 °C for 16 hours to remove the GST tag. The complex was purified away from the GST by cation exchange chromatography using a 5 mL Heparin HP column (GE Healthcare) with a linear salt gradient of 0.1 – 1 M sodium chloride in 20 mM HEPES-KOH pH 7.5. Fractions containing the complex in a 2:1 molar ratio, Vps75:Rtt109, were pooled and dialysed into 20 mM HEPES-KOH pH 7.5, 0.3 M sodium chloride, 1 mM EDTA, concentrated and stored at -80 °C.

Rtt109 was expressed on its own from a pET28a derived vector, a kind gift from Paul Kaufman. The N-terminal 6xhis tagged Rtt109 was purified essentially the same way as Nap1/Vps75/Asf1 except a Heparin HP column was used for ion exchange chromatography (GE Healthcare).

Sulfhydryl reactive crosslinking

For probing the H3-H4 conformation when in complex with Asf1, Vps75 and Nap1, H3 K115C tetramer-chaperone complexes (cysteine null chaperones) were made up in 20 mM HEPES-KOH pH 7.5, 0.2 M sodium chloride, 1 mM EDTA at a concentration of 1 µM histone tetramer. 1,3-Propanediyl bismethanethiosulfonate (MTS-3-MTS) (Toronto Research Chemicals) was made up in dimethyl sulfoxide (DMSO) to a concentration of 25 µM. 48 µL of 1 µM complex was added to 2 µL of MTS-3-MTS to achieve a 1:1 ratio of crosslinker to histone tetramer, and the crosslinking reaction allowed to proceed for 3 minutes, at which point MTSL was added to a concentration of 200 µM to quench any unreacted cysteine residues. Quenching proceeded for one minute before samples were subjected to non-reducing SDS-PAGE.

To look at binding of Asf1 to the crosslinked tetramer, Asf1 was mixed at 20 µM with an equal stoichiometry of pre-crosslinked tetramer (10 µM) in a total volume of 30 µL. Binding was allowed to take place for 1 hour at 25 °C and complexes separated by filtration chromatography. Fractions were analysed by SDS-PAGE and bands quantified by the software AIDA (Raytest).

For crosslinking of endogenous histones, purified complexes were reduced with 20 mM DTT before microdialysis for 12-14 hours against E-buffer (20 mM HEPES-KOH pH 7.5, 150 mM NaCl, 10 % glycerol, 0.05 % Tween20). The reduced samples (normalized to total H3K115C-myc as probed by immuno-blotting) were diluted to 40 µL in E-buffer and subjected to crosslinking for two hours at 26°C by the addition of 0.064, 0.25 or 1.0 mM

Cu(II):1,10-phenanthroline$_3$ (Melford Laboratories Ltd) in 50 % dimethylsulfoxide. The reactions were quenched by the addition of 200 μM MTSL and 10 mM EDTA before separation by SDS-PAGE and visualization by immuno-blotting using and anti-myc antibody (Upstate).

Preparation of crosslinked tetramers for tetrasome assembly and histone acetyltransferase assays

For the tetrasome assembly reactions, aliquots of reduced histone tetramer containing the H3 K115C mutation were thawed, diluted with 1 M sodium chloride, 20 mM HEPES pH 7.5 and 1 mM EDTA to 15 μM (tetramer) and crosslinked with a 1:1 stoichiometry of bis-maleimidoethane (BMOE) (Thermo Fisher) that had been dissolved in DMSO. At this higher concentration of tetramer the reaction was more efficient resulting in ~80 % crosslinking. The final concentration of dimethyl sulfoxide was kept below 4 % total volume. BMOE was used instead of MTS-3-MTS as it forms a thioether, rather than a disulphide, and is thus cannot be reduced by DTT. Crosslinking proceeded for 3 minutes and was quenched with 10 mM DTT. Crosslinked tetramer was used directly in tetrasome assembly.

For acetyltransferase assays, H3 K115C tetramer crosslinking was monitored over time and analysed by SDS-PAGE to asses the extent of the crosslinking. The time point that was closest to an equimolar ratio of crosslinked to uncrosslinked tetramer was used in the competition assay.

Preparation and validation of spin labeled histone-histone chaperone complexes

Cysteine null mutants of Nap1 and Vps75 and spin labeled histone tetramer were buffer exchanged into refolding buffer made up with deuterium oxide and 0.8 M sodium chloride using multiple rounds of concentration and dilution using Amicon Ultra centrifugal concentrators (Millipore) and finally concentrated to 200 μM Nap1/Vps75 dimer, 200 μM histone tetramer. 25 μL of spin labeled histone tetramer was added to 25 μL of concentrated chaperone to achieve the correct binding stoichiometry. To this 50 μL of 97 % D8-glycerol was added (Cambridge Isotope Laboratories), to give a final sodium chloride concentration of 0.4 M and a final complex concentration of 50 μM (histone tetramer or chaperone dimer), mixed well and stored at -80 °C until distance extraction by EPR. Complexes containing Asf1 were prepared the same way except buffer exchange was carried out with 1 M sodium chloride resulting in a final concentration of 0.5 M in 50 % D8-glycerol. The salt concentration of the samples was within toleration of the complex shown by gel filtration. To further validate that complex formation was occurring after distance extraction 10 μL of the sample was subject to gel filtration chromatography (Superdex S200 PC 3.2/30, GE Healthcare) under identical buffer conditions under which the PELDOR experiment was carried out (20 mM HEPES-KOH, 1 mM EDTA, 50 % glycerol, 0.4/0.5 M NaCl). Co-elution of histones and chaperone was observed in all cases.

PELDOR data analysis

The experimentally obtained time domain trace is processed so as to remove any unwanted intermolecular couplings, which is called the background decay. Tikhonov regularisation is then used to simulate time trace data that give rise to distance distributions, P(r), of different peak width depending on the regularisation factor, alpha. The alpha term used was judged

by reference to a calculated L-curve. The L curve is a parametric plot that compares smoothness of the distance distribution to the mean square deviation. The most appropriate alpha term to be used is at the inflection of the L curve, since this provides the best compromise between smoothness (artifact suppression) and fit to the experimental data. PELDOR data was analysed using the DeerAnalysis 2006 software package (Jeschke et al., 2006). The dipolar coupling evolution data was corrected for background echo decay using a homogeneous three-dimensional spin distribution. The starting time for the background fit was optimised to give the best fit Pake pattern in the Fourier transformed data and the lowest root mean square deviation background fit.

The Pake pattern can allow distance determination using the following equation,

$$f_{Dip}(r,\theta) = \frac{\mu_B^2 g_A g_B \mu_0}{2\pi h} \cdot \frac{1}{r_{AB}^3}(3\cos^2\theta - 1)$$

where θ is the angle between the spin-spin vector r and the direction of the applied magnetic field, μ_B is the Bohr magneton, μ_0 is the permeability of free space, g_A and g_B are the g values for the two spin labels A and B, and r is the spin-spin distance, assuming the exchange coupling constant can be neglected. If a resolved perpendicular turning point feature is observed in the spectrum a mean distance can be inferred.

Strain construction

A cysteine at H3 K115 was introduced using PCR based quick-change method for crosslinking studies. Plasmid pNOY439 (*CEN6 ARS4 TRP1* MYC-*HHT2-HHF2*) (Schermer et al., 2005) was used as a template to generate pNOY439-H3K115C. To generate the HHT2-Myc tag strain, WZY42 strain (Zhang et al., 1998) (Δ *hht1-hhf1*, Δ *hht2-hhf2*, and Ycp50 carrying *HHT1-HHF1*) was transformed with the plasmid pNOY439-H3K115C , driving the expression of Myc-Hht2K115C and untagged Hhf2 (H4) from their endogenous promoter. Subsequently, loss of Ycp50 was selected for on 5-FOA resulting in ectopic myc tagged H3 K115C being the only source of histone H3. At this stage TAP tag was introduced at the C-terminus of the genomic copies of three histone chaperones (ASF1, NAP1 and VPS75) by transforming PCR products with the tag. The tagging methodology has been detailed elsewhere (Longtine et al., 1998). The primers used in the construction of the strains and quick-change to introduce K115C mutation in HHT2 (pNOY439) will be provided on request.

Preparation of non-tetramerising mutants, nucleosome assembly and crosslinking reactions

Histone H3 containing mutations C110E and H113A were refolded with wild-type H4 by dialysis from 7 M guanidinium chloride, 20 mM HEPES-KOH pH7.5 to 20 mM HEPES-KOH pH7.5, 200 mM sodium chloride, 2 mM DTT, and subjected to gel filtration chromatography in the same buffer minus the DTT. Fractions spanning peak traces were analysed by SDS-PAGE. Fractions containing the refold complexes were pooled, concentrated and stored at -80°C until use. Non-tetramerising histones containing the H3 K115C mutation were purified in the same way, except that 20 mM DTT was added before gel filtration in ensure complete reduction of cysteine residues.

Nucleosomes were assembled by salt dialysis. 147 base pair DNA, comprising the 601 sequence (Thastrom et al., 1999), was mixed at a final concentration of 2 μM together with 4 μM H3-H4 dimer and 4 μM H2A-H2B dimer in Refolding Buffer. These were subjected to continuous salt dialysis (Thastrom et al., 2004), reaching a final concentration of 100 mM sodium chloride. Assembly of nucleosomes was analysed by native PAGE electrophoresis.

Crosslinking of non-tetramerising mutants containing the H3 K115C mutation was carried out at 2 μM histone dimer (1 μM tetramer) in the presence and absence of 2 μM Nap1 (1 μM Nap1 dimer) in 20 mM HEPES-KOH pH 7.5, 0.2 M sodium chloride. Cu(II):1,10-phenanthroline$_3$ (derived from copper sulphate) was added to a final concentration of 4 μM and the reaction was allowed to proceed for 30 seconds before quenching with 200 μM MTSL and 10 mM EDTA.

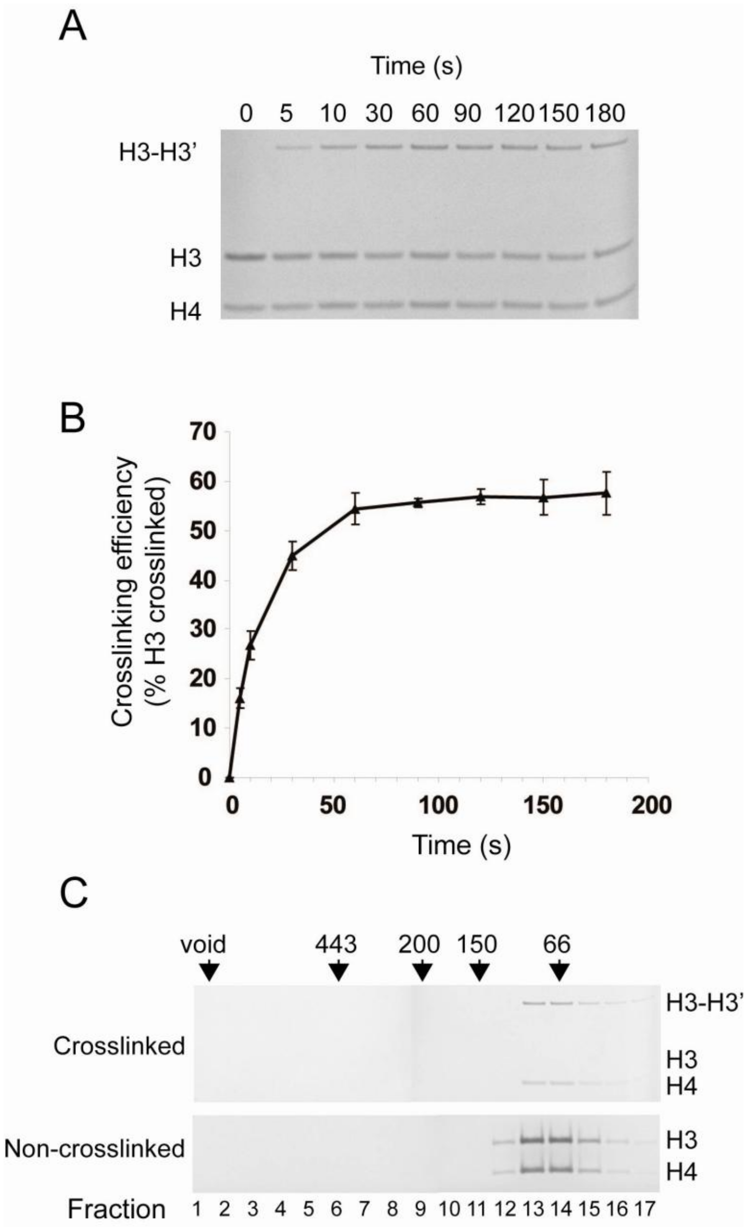

Figure S1. Site Directed Crosslinking of the Histone Tetramer, Related to Figure 2

(A) H3 K115C tetramer (1 μM) was subjected to an equimolar amount of the crosslinker MTS-3-MTS and crosslinking was monitored over a period of 180 seconds. Time points indicated were resolved on a non-reducing SDS-PAGE gel. (B) Quantification of the time points shown in (A) and plotted as a function of the percentage of H3 crosslinked over time. Error bars represent the standard deviation of three independent experiments. (C) H3 K115C crosslinked tetramer was subjected to analytical gel filtration chromatography. Elution of tetramer crosslinked at position K115C displayed an identical elution profile to that of non-crosslinked tetramer, indicating that specific crosslinking across the dyad interface is occurring.

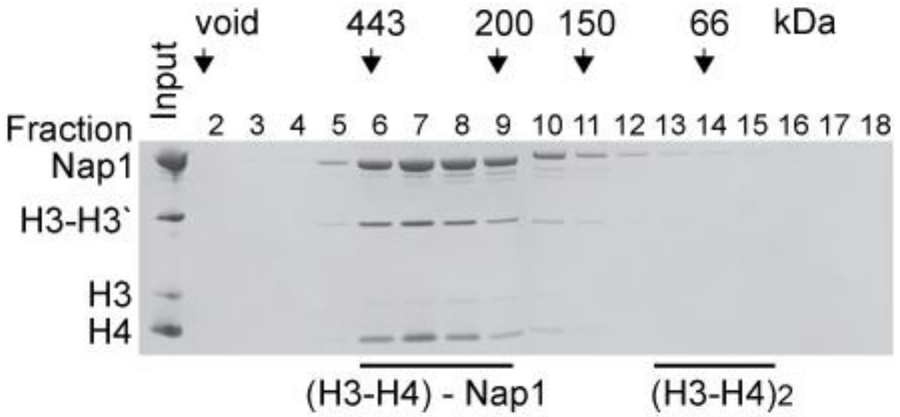

Figure S2. Crosslinking Does Not Reduce the Association of Nap1 with Histones H3 and H4, Related to Figure 2

To determine whether crosslinking at position H3K115C has an effect on Nap1 binding to the $(H3\text{-}H4)_2$-tetramer pre-crosslinked tetramer was incubated with Nap1 and allowed to equilibrate at 4ºC before separation by gel filtration chromatography. The pre-crosslinked tetramer co-eluted with Nap1 in the same fractions as tetramer crosslinked in complex with Nap1 (compare to Figure 2C in the main text) suggesting crosslinking at H3K115C has little or no effect on the H3H4-Nap1 interaction.

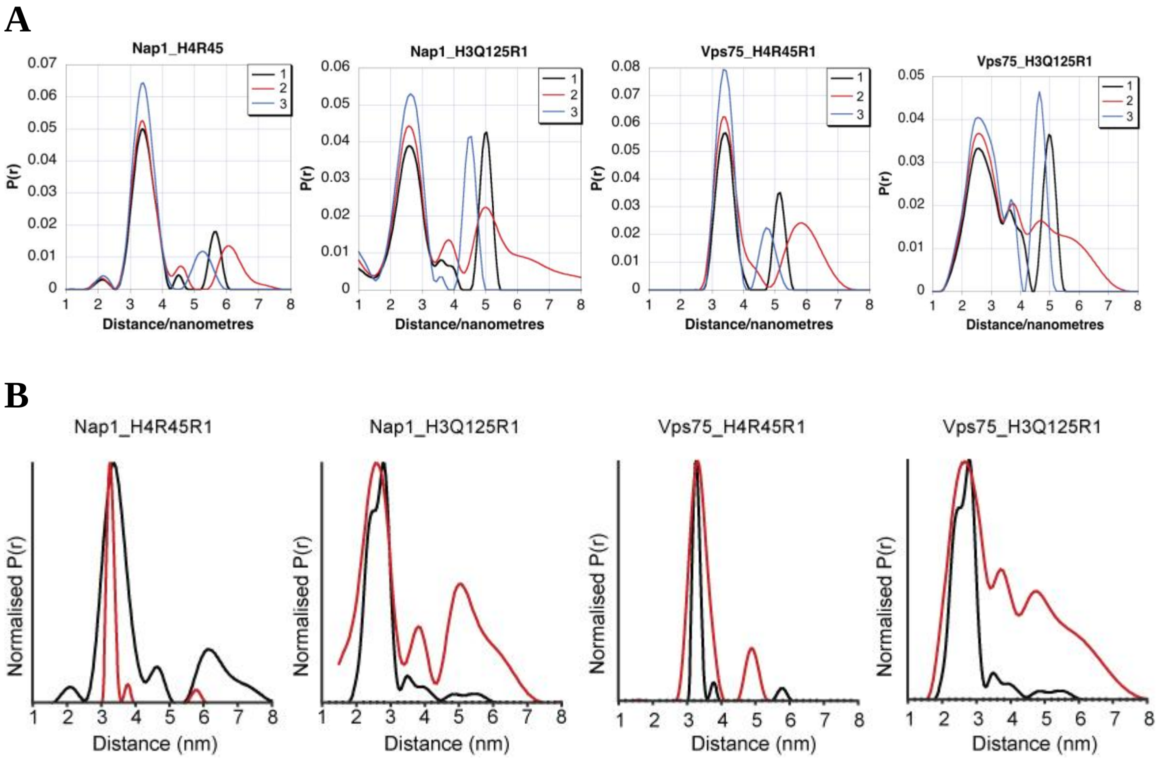

Figure S3. Validation of Major PELDOR Distributions and Comparison to the Histone Octamer, Related to Figure 3

(A) Background correction parameters can result in apparent minor distance distributions. Overlaid distance distribution data obtained under three different background correction conditions (1, 2, and 3) for chaperone-histone complexes detailed in Figure 3. Processing conditions 1 (black line): background correction starts at short time (start time 320 ns) and all the time data collected during the experiment is used in the processing (end time 3160 ns). Processing conditions 2 (red line): background correction starts at longer time (start time 2000 ns) and continues to the same end time of 3160 ns (Red data). Processing conditions 3 (blue line): background correction starts at short time (start time 320 ns) and continues to a truncated end time of 2260 ns, instead of 3160 ns. Note that distance distributions for background correction artifacts (above 4Å) vary with the correction parameters, yet the main distance distributions are robust. (B) Long-range distances extracted from spin labeled H3-H4 in the presence of histone chaperones (Nap1 or Vps75) compared to data obtained using the same spin labeling positions where the tetramer is in the presence of H2A-H2B, and thus forms a histone octamer (Bowman et al., 2010). Distance distribution data for H4R45R1, H3Q125R1, H4R45R1, and H3Q125R1 as part of an octamer (black line) or in the presence of chaperone (red line).

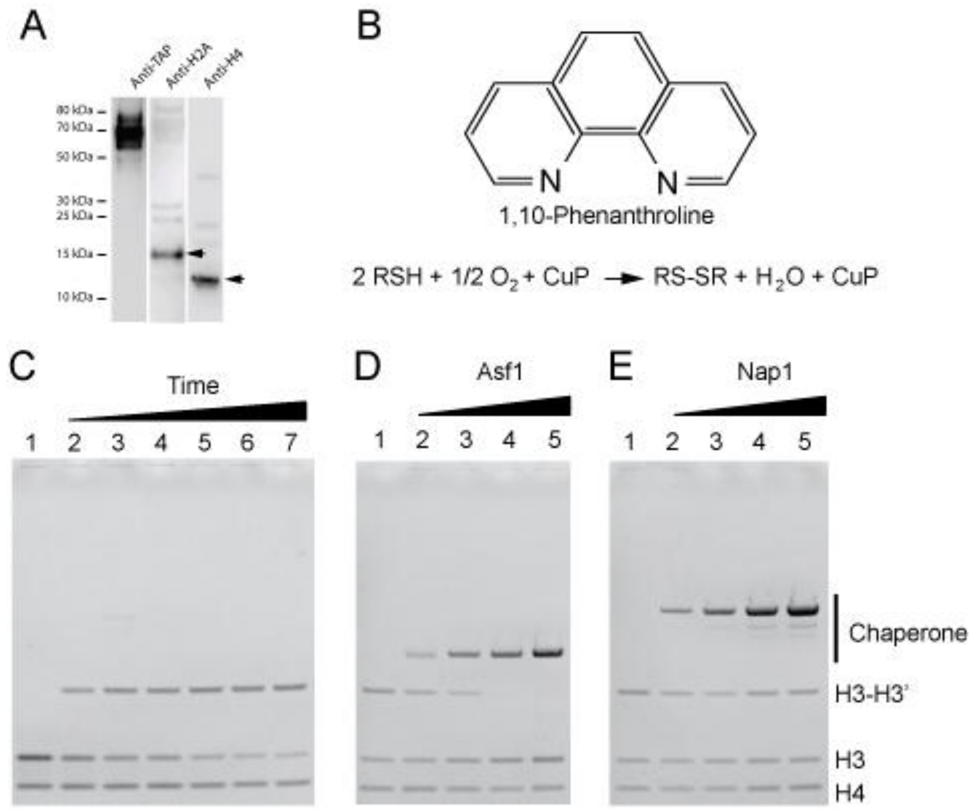

Figure S4. Development of a Crosslinking Approach to Study the Conformation of Native Histones in Association with Chaperones, Related to Figure 5

(A) Nap1 from yeast co-purifies with H2A-H2B and H3-H4. TAP-tagged Nap1 was purified from unsynchronised mid-log phase cells and purified by IgG and calmodulin affinity resins. Immuno blot analysis using antibodies specific to H2A and H4 identified bands with a comparable molecular weights when compared to protein standards (arrows). White lines indicate lanes from the same blot, but probed with the corresponding antibody. Anti-TAP and anti-H2A lanes were equally loaded, anti-H4 contained a 5-fold excess. (B-E) Cu(II)- 1,10-Phenanthroline3 (CuP) as a method for inducing a native disulphide bond between H3 K115C residues within the histone tetramer. (B) Chemical structure of 1,10-Phenanthroline and the chemical equation governing the catalysis of sulfhydryl oxidation through chelation of copper(II). Dissolved molecular oxygen is thought to act as a proton acceptor in the oxidation of thiol groups (RSH) producing a disulphide (RS-SR) and water (Kobashi, 1968). (C) Disulphide bond formation at position H3 K115C on the histone tetramer (1 μM) was monitored as a function of time at a CuP concentration of 4 μM: lane 1, non-crosslinked input; lane 2, 5 seconds; lane 3, 30 seconds; lane 4, 1 minute; lane 5, 5 minutes; lane 6, 20 minutes; lane 7, 60 minutes. (D) Disulphide bond formation was inhibited in the presence of Asf1: lane 1, tetramer alone; lane 2, 0.5 mM Asf1; lane 3, 1 mM Asf1; lane 4, 1.5 mM Asf1; lane 5, 2 mM Asf1. (E) In the presence of Nap1 disulphide bond formation was not affected: lane 1, tetramer alone; lane 2, 0.5 mM Nap1; lane 3, 1 mM Nap1; lane 4, 1.5 mM Nap1; lane 5, 2 mM Nap1. In both (D) and (E) disulphide bond formation was induced by the addition of CuP to a concentration of 4 μM, and allowed to proceed for 30 seconds before quenching with 10 mM EDTA and 200 mM MTSL.

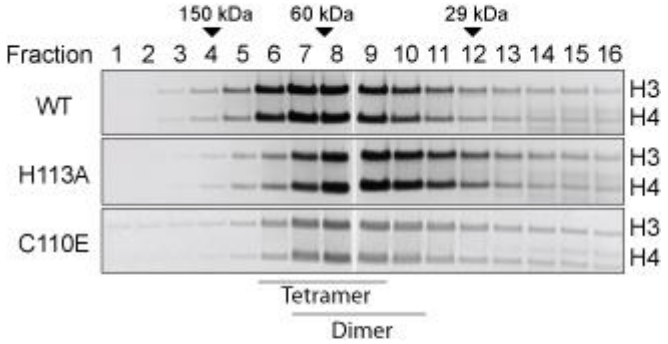

Figure S5. H3 H113A and H3 C110E Mutations Shift the Tetramer-Dimer Equilibrium of H3-H4 towards the Dimer, Related to Figure 6

SDS-PAGE analysis of gel filtration fractionation shows a slight shift in elution volume from H3-H4 carrying the non-tetramerising mutants H3 H113A and C110E to wild-type (WT) under physiologically relevant salt concentrations (0.2 M sodium chloride). The four peak fractions are indicated for WT (tetramer) and mutants (dimer) below.

Supplemental References

Andrews, A.J., Chen, X., Zevin, A., Stargell, L.A., and Luger, K. (2010). The Histone Chaperone Nap1 Promotes Nucleosome Assembly by Eliminating Nonnucleosomal Histone DNA Interactions. Molecular Cell *37*, 834-842.

Andrews, A.J., Downing, G., Brown, K., Park, Y.J., and Luger, K. (2008). A thermodynamic model for Nap1-histone interactions. Journal of Biological Chemistry *283*, 32412-32418.

Bowman, A., Ward, R., El-Mkami, H., Owen-Hughes, T., and Norman, D.G. (2010). Probing the (H3-H4)2 histone tetramer structure using pulsed EPR spectroscopy combined with site-directed spin labelling. Nucleic Acids Research *38*, 695-707.

Jeschke, G., Chechik, V., Ionita, P., Godt, A., Zimmermann, H., Banham, J., Timmel, C.R., Hilger, D., and Jung, H. (2006). DeerAnalysis2006 - A comprehensive software package for analyzing pulsed ELDOR data. Applied Magnetic Resonance *30*, 473-498.

Longtine, M.S., McKenzie, A., 3rd, Demarini, D.J., Shah, N.G., Wach, A., Brachat, A., Philippsen, P., and Pringle, J.R. (1998). Additional modules for versatile and economical PCR-based gene deletion and modification in Saccharomyces cerevisiae. Yeast *14*, 953-961.

Luger, K., Rechsteiner, T.J., Flaus, A.J., Waye, M.M.Y., and Richmond, T.J. (1997). Characterization of nucleosome core particles containing histone proteins made in bacteria. Journal of Molecular Biology *272*, 301-311.

Park, Y.J., Sudhoff, K.B., Andrews, A.J., Stargell, L.A., and Luger, K. (2008). Histone chaperone specificity in Rtt109 activation. Nature Structural and Molecular Biology *15*, 957-964.

Schermer, U.J., Korber, P., and Horz, W. (2005). Histones are incorporated in trans during reassembly of the yeast PHO5 promoter. Mol Cell *19*, 279-285.

Thastrom, A., Lowary, P.T., Widlund, H.R., Cao, H., Kubista, M., and Widom, J. (1999). Sequence motifs and free energies of selected natural and non-natural nucleosome positioning DNA sequences. J Mol Biol *288*, 213-229.

Thastrom, A., Lowary, P.T., and Widom, J. (2004). Measurement of histone-DNA interaction free energy in nucleosomes. Methods *33*, 33-44.

Zhang, W., Bone, J.R., Edmondson, D.G., Turner, B.M., and Roth, S.Y. (1998). Essential and redundant functions of histone acetylation revealed by mutation of target lysines and loss of the Gcn5p acetyltransferase. EMBO J *17*, 3155-3167.
